# Pharmacological properties, molecular mechanisms and therapeutic potential of ginsenoside Rg3 as an antioxidant and anti-inflammatory agent

**DOI:** 10.3389/fphar.2022.975784

**Published:** 2022-09-05

**Authors:** Jing Wang, Li Zeng, Ying Zhang, Wenxiu Qi, Ziyuan Wang, Lin Tian, Daqing Zhao, Qibiao Wu, Xiangyan Li, Tan Wang

**Affiliations:** ^1^ Department of Respiratory, The Affiliated Hospital to Changchun University of Chinese Medicine, Changchun, China; ^2^ State Key Laboratory of Quality Research in Chinese Medicines, Faculty of Chinese Medicine, Macau University of Science and Technology, Macau, China; ^3^ Jilin Ginseng Academy, Changchun University of Chinese Medicine, Changchun, China; ^4^ Guangdong-Hong Kong-Macao Joint Laboratory for Contaminants Exposure and Health, Guangzhou, China

**Keywords:** ginsenoside Rg3, oxidative stress, inflammation, molecular mechanism, therapeutic effect

## Abstract

Inflammation and oxidative stress lead to various acute or chronic diseases, including pneumonia, liver and kidney injury, cardiovascular and cerebrovascular diseases, metabolic diseases, and cancer. Ginseng is a well-known and widely used ethnic medicine in Asian countries, and ginsenoside Rg3 is a saponin isolated from *Panax ginseng C. A. Meyer, Panax notoginseng,* or *Panax quinquefolius L.* This compound has a wide range of pharmacological properties, including antioxidant and anti-inflammatory activities, which have been evaluated in disease models of inflammation and oxidative stress. Rg3 can attenuate lung inflammation, prevent liver and kidney function damage, mitigate neuroinflammation, prevent cerebral and myocardial ischemia–reperfusion injury, and improve hypertension and diabetes symptoms. The multitarget, multipathway mechanisms of action of Rg3 have been gradually deciphered. This review summarizes the existing knowledge on the anti-inflammatory and antioxidant effects and underlying molecular mechanisms of ginsenoside Rg3, suggesting that ginsenoside Rg3 may be a promising candidate drug for the treatment of diseases with inflammatory and oxidative stress conditions.

## 1 Introduction

Ginseng, the root of *Panax ginseng C.A. Meyer*, has been used for 2000 years in ethnomedicine in Asia to treat various diseases (Lee et al., 2020a). The roots, leaves, and fruits of ginseng plants have been extensively used as herbal medicines. The antioxidant activity of ginseng is exploited to treat cardiovascular diseases (Hyun et al., 2022). Interestingly, red ginseng has better antioxidant activity and is used to treat neurodegenerative diseases, gastroenteritis, liver damage, diabetes, and aging (Lee et al., 2017a). The Rg3 content of fresh ginseng is relatively low, and the content in ginseng after processing or enzymatic transformation accounts for 0.37–1.13% of the total saponins (Chang et al., 2009; Kim et al., 2013). Red ginseng has undergone steaming treatment. During this process, some ginsenosides with lower polarity are produced. The Rg3 content increases with increasing steaming batches. After nine steam cycles, the Rg3 content in the total saponins was 15–18% (Lee et al., 2012a; Jeong et al., 2017). According to the same process used to make red ginseng roots, Chen *et al.* steamed ginseng leaves, and Rg3 increased 8.6-fold (Chen et al., 2020). These findings suggest that Rg3 is one of the primary ginsenosides in red ginseng.

**FIGURE 1:**
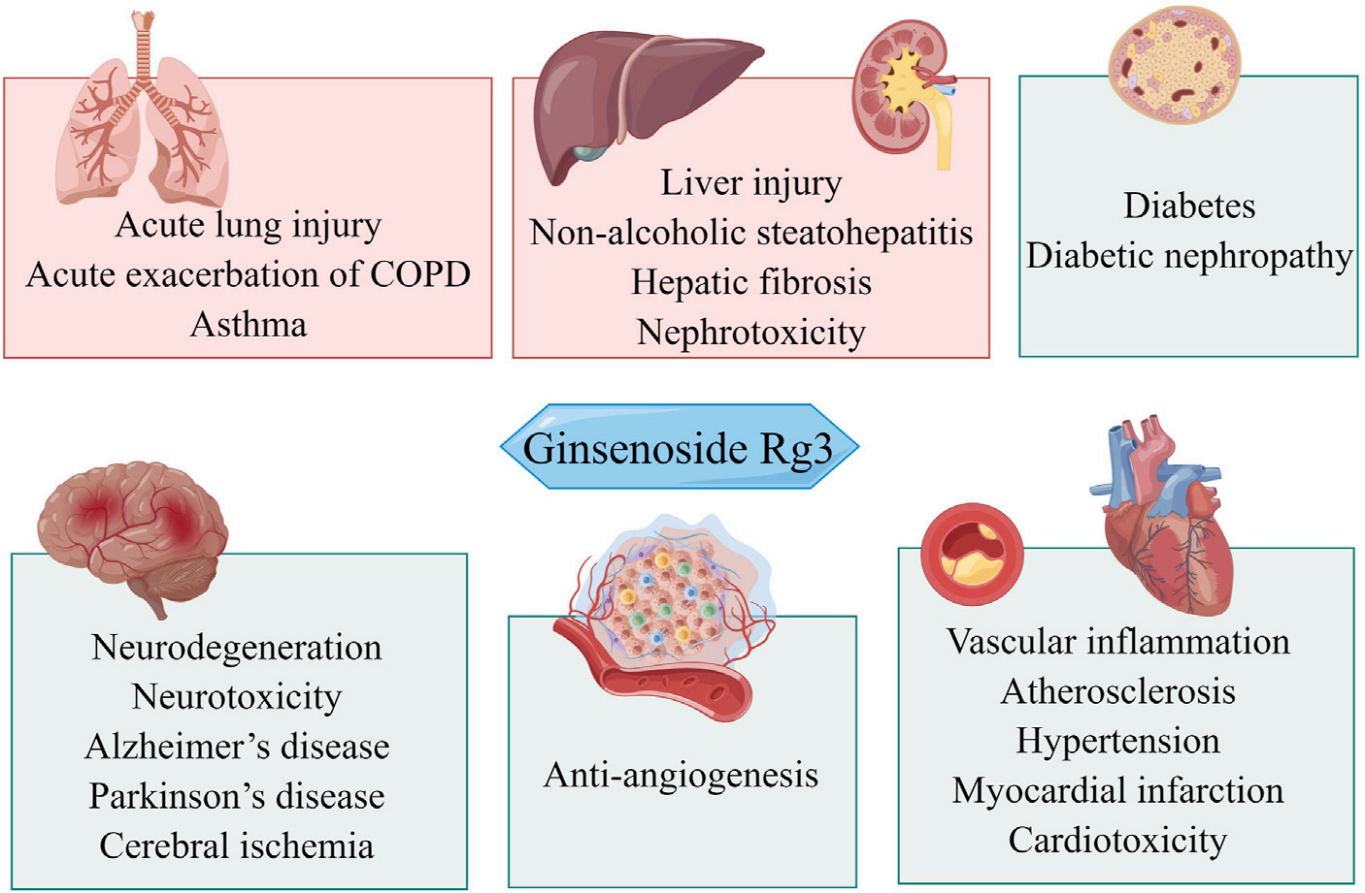
Overview of the potential therapeutic effects of Rg3 on different organ systems.

This work aimed to understand how Rg3 regulates oxidative stress and inflammation to elicit pharmacological effects and to review the literature on the therapeutic use of Rg3 based on antioxidant and anti-inflammatory effects. We briefly introduced the biological functions of Rg3 in response to inflammation and oxidative stress. We then summarized the effects of Rg3 on neurological disorders, cardiovascular diseases, cancer, lung diseases, diabetes, and digestive system diseases (Figure 1).

## 2 Methodology

A literature search was performed in PubMed from August 1998 to January 2022, and the last search date was 30 April 2022. The search term was “Ginsenoside Rg3”. A secondary search was conducted by screening the list of articles that met the inclusion criteria. The keywords were inflammation, oxidative stress, antioxidant, and anti-inflammatory. The obtained articles were screened, irrelevant studies were excluded, and eligible articles were sorted and classified. Finally, we organized the tables, wrote the text, and made figures to summarize the application of Rg3 antioxidant and anti-inflammatory effects.

## 3 Overview of oxidative stress and inflammation

Reactive oxygen species (ROS), including superoxide, hydroxyl radicals, and hydrogen peroxide (H_2_O_2_), are necessary for cell growth, differentiation, responses to external stimuli, and other processes. ROS are primarily produced in the mitochondria through the respiratory chain and the nicotinamide adenine dinucleotide phosphate (NADPH) oxidase system. In addition to mitochondria, cytochrome P450 2E1 (CYP2E1), cyclooxygenase-2 (COX-2), and lipoxygenases in the plasma membrane and cytosol produce ROS. ROS are subsequently eliminated by superoxide dismutase (SOD), catalase (CAT), glutathione peroxidase (GSH-px), and nonenzymatic scavenging factors, such as glutathione (GSH) and vitamin C, to maintain redox homeostasis (Ramos-Tovar and Muriel, 2020). In response to ROS accumulation, peroxidase production activates the NF-E2-related factor 2 (Nrf2)/heme oxygenase 1 (HO-1) pathway (Kobayashi and Yamamoto, 2005). Lipid oxidation produces toxic metabolic compounds, hydroperoxides, and hydroxides; they aggravate the formation of atherosclerosis and liver injury. Malondialdehyde (MDA) is an end product of lipid oxidation used as a biomarker for assessing oxidative damage (Ayala et al., 2014; Silva et al., 2022). Nitric oxide synthase (NOS) catalyzes the production of nitric oxide (NO) and citrulline using oxygen and l-arginine as substrates. There are three isoforms of NOS. Endothelial NOS (eNOS) and neuronal NOS (nNOS) are expressed in the normal state. NO produced by eNOS is critical for maintaining vascular homeostasis (Balligand et al., 2009). Inducible NOS (iNOS) is induced after injury (Brown, 2007). iNOS-derived NO reacts with superoxide anions to form peroxynitrite, leading to oxidative stress related to inflammatory neurodegeneration, endothelial dysfunction, and liver and kidney injury (Jaeschke et al., 2002; Landmesser et al., 2003; Heemskerk et al., 2009; Incalza et al., 2018).

Oxidative stress and inflammation occur concomitantly. The accumulation of ROS can induce inflammation. In turn, the inflammatory response triggers oxidative stress. In the inflammatory response, inflammatory factors activate Toll-like receptor (TLR) signaling receptors in neutrophils and macrophages, and inflammatory mediators are released in large quantities. This process leads to oxygen consumption, which is produced through the NADPH pathway, and the accumulation of oxygen free radicals causes oxidative stress (Furman et al., 2019). When cells are damaged by oxidative stress, ROS accumulate in large quantities and activate TLR receptors in Kupffer cells and macrophages, thus increasing proinflammatory mediators such as the release of interleukin-1β (IL-1β) and IL-6 (Yoon et al., 2015; Ramos-Tovar and Muriel, 2020).

## 4 Antioxidant and anti-inflammatory effects and molecular mechanisms of Rg3

In response to oxidative stress, Rg3 inhibits ROS accumulation and MDA content, improves mitochondrial function, and increases the activity of antioxidant enzymes, including SOD, GSH, GSH-px, and CAT, to promote oxygen free radical metabolism. Rg3 responds to oxidative stress by regulating Nrf2/HO-1 and sirtuin 1 (SIRT1) (Xing et al., 2017; Kee and Hong, 2019; Ren et al., 2021). Rg3 inhibits iNOS and NO release to reduce peroxynitrite formation; interestingly, it promotes eNOS-derived NO to protect vascular endothelial cells (Singh et al., 2018).

In lipopolysaccharide (LPS)-mediated inflammation models, Rg3 decreased the expression of COX-2 and prostaglandin E2 (PGE2) and the secretion of tumor necrosis factor-α (TNF-α), IL-1β, and IL-6 (Shin et al., 2013; Lee et al., 2017b; Kee and Hong, 2019). Inhibitory NLRP3 inflammasome activation and NLRP3-apoptosis-associated speck-like protein (ASC) interaction were observed in an LPS-induced mouse model after treatment with Rg3 (Kang et al., 2018; Shi et al., 2020). The mechanisms of response to inflammation relate to inhibition of the nuclear factor-κB (NF-κB) pathway, including upstream TLR4/myeloid differentiation primary response 88 (MyD88) and downstream COX-2 expression (Kang et al., 2018; Candelario-Jalil et al., 2022). Rg3 upregulates SIRT1 to inhibit the NF-κB pathway, contributing to anti-inflammatory activity (Ren et al., 2021). Downregulation of the phosphatidylinositol-3-kinase (PI3K)/Akt and p38 mitogen-activated protein kinase (MAPK) pathways by Rg3 suppressed inflammation directly or by crosstalk with the NF-κB pathway (Hou et al., 2017; Yang et al., 2018; Kee and Hong, 2019).

### 4.1 Pulmonary diseases

Inflammation is present in various pulmonary diseases. Persistent inflammation in the airway and lung tissue is involved in the pathogenesis of chronic lung diseases such as chronic obstructive pulmonary disease (COPD), asthma, and pulmonary fibrosis (Racanelli et al., 2018). Acute lung disease is often accompanied by acute inflammation, including acute lung injury (ALI), pneumonia, and acute exacerbation of COPD (AECOPD) (Robb et al., 2016). Inflammation promotes the accumulation of oxidative stress products. These findings suggest that anti-inflammation is a critical step in pulmonary disease treatment (Domej et al., 2014). In animal models of chemical-induced ALI, Rg3 inhibited cytokines and increased the content of antioxidant enzymes (SOD, CAT, and GSH) to reduce pulmonary inflammation and neutrophil infiltration (Wang et al., 2016; Yang et al., 2018). The anti-inflammatory effect of Rg3 was also observed in the AECOPD mouse model, and these effects were related to the inhibition of the PI3K/Akt pathway (Yang et al., 2018; Guan et al., 2020). In an airway inflammation model stimulated by Asian sand dust, upregulation of mucin (MUC)5AC and MUC5B stimulates mucus secretion; significantly, mucus cross-linking exacerbates pulmonary disease (Dunican et al., 2021). Rg3 decreased mucin gene expression through the NF-κB pathway (Shin et al., 2021). Regarding asthma, Rg3 inhibited p65 phosphorylation, and COX-2 inhibited the secretion of chemokine ligands, ILs, and TNF-α. Rg3 exerts its antioxidant effect through the Nrf2/HO-1 pathway to decrease ROS generation. Anti-inflammatory and antioxidant effects have been used to treat asthma models *in vitro* and *in vivo* (Lee et al., 2016; Huang et al., 2021). In summary, Rg3 exerts anti-inflammatory effects to alleviate lung damage (Table 1).

**TABLE 1:** Summary of Rg3 in pulmonary diseases.

Diseases	Model	Treatment	Outcome	Mechanism	Ref
**ALI**	Male SD rats injected subcutaneously with 60 mg/kg omethoate	5, 10, and 20 mg/kg Rg3 via tail vein	MDA↓, SOD↑, CAT↑, GSH↑, MPO↓, TNF-α↓	Improved lung inflammation and neutrophil infiltration by antioxidant function	Wang et al. (2016)
**ALI**	RAW264.7 cells stimulated with 2 μg/mL LPS	25, 50, or 100 μg/mL Rg3	MPO↓, TNF-α↓, IL-1β↓, IL-6↓, IL-10↑, TGF-β↓, PI3K/Akt/mTOR↓, p-MerTK↑	Decreased MerTK to mediated activation of PI3K/Akt/mTOR pathway	Yang et al. (2018)
	C57BL/6 mice and MerTK^−/−^ C57BL/6 mice with 10 μg LPS intranasally	10, 20, and 30 mg/kg Rg3 intraperitoneally			
**AECOPD**	BEAS–2B cells with cigarette smoke	10, 20 and 40 μM 20 (S)-Rg3	Neutrophil migration↓, IL-6↓, p-PI3K↓, p-Akt↓	Reduced the migration of neutrophils through downregulation of PI3K/Akt pathway	Guan et al. (2020)
	BALB/c mice exposured to cigarette smoke for 14 weeks then infected with non–typeable *Haemophilus* inflenza	10, 20, 40 mg/kg 20 (S)-Rg3 intragastrically			
**Airway inflammatory disease**	BEAS-2B cells stimulated with 50 or 100 μg/mL of Asian sand dust for 48 h	50 μg/mL Rg3	MUC5AC↓, MUC5B↓, NF-κB↓	Inhibited mucin gene expression and protein through NF-κB pathway	Shin et al. (2021)
**Asthma**	10 ng/mL IL-1β induced A549 cells	900 nM Rg3	COX-2↓, IL-4↓, TNF-α↓, eotaxin↓, p-p65↓	Inhibited NF-κB p65 activity for anti-inflammatory effect	Lee et al. (2016)
	Human asthmatic airway epithelial tissues	50 μM Rg3			
**Asthma**	BEAS-2B cells with 10 ng/mL IL-4/TNF-α	3, 10 and 30 μM Rg3	Eotaxin↓, Eotaxin-2↓, TNF-α↓, IL-5↓, IL-4↓, IL-13↓, IL-6↓, and IFN-γ↑, COX-2↓, ICAM-1↓, Nrf2↑, HO-1↑, MDA↓, GSH↑, ROS↓	Inhibited the oxidative stress and inflammation via Nrf2/HO-1 pathway	Huang et al. (2021)
	Female BALB/c mice with intraperitoneal injection of 50 μg ovalbumin	Intraperitoneal injection of 10 mg/kg Rg3			

ALI, acute lung injury; SD, rat, Sprague–Dawley rat; MerTK, mer tyrosine kinase; LPS, lipopolysaccharide; MDA, malondialdehyde; SOD, superoxide dismutase; CAT, catalase; GSH, glutathione; MPO, myeloperoxidase; TNF-α, tumor necrosis factor-α; IL, interleukin; TGF-β, transforming growth factor-β; PI3K/Akt/mTOR, phosphatidylinositol-3-kinase/Akt/mammalian target of rapamycin; AECOPD, acute exacerbation chronic obstructive pulmonary disease; MUC, mucin; NF-κB, nuclear factor-κB; COX-2, cyclooxygenase-2; IFN-γ, interferon-γ; ICAM-1, intercellular adhesion molecule-1; Nrf2, NF-E2-related factor 2; HO-1, heme oxygenase 1; ROS, reactive oxygen species.

### 4.2 Liver and kidney injury

Several studies have demonstrated that Rg3 downregulates abnormally elevated alanine aminotransferase (ALT) and aspartate aminotransferase (AST) to improve liver function in models of drug-induced or inflammation-mediated liver injury (Kang et al., 2007; Zhou et al., 2018). In addition, Rg3 alleviated acetaminophen-mediated liver tissue apoptosis by activating the PI3K/Akt pathway (Zhou et al., 2018). In a mouse model of acetaminophen-mediated/induced liver injury, Rg3 reduced GSH consumption and inhibited CYP2E1 overexpression to combat oxidative stress and hepatotoxicity (Zhou et al., 2018; Gao et al., 2021). Rg3 decreased thiobarbituric acid (TBA) and iNOS to mitigate oxidative damage in the livers and kidneys of LPS-treated rats (Kang et al., 2007). Rg3 also protected against acute liver and kidney function injury caused by chemotherapeutic agents. Chemotherapy containing cis-platinum (DDP) causes liver and kidney damage, and several studies found that Rg3 treatment significantly protected against hepatotoxicity and nephrotoxicity. ALT, AST, blood urea nitrogen (BUN), and creatinine (CRE), which represent liver and kidney function, decreased after treatment with Rg3 in a DDP-induced mouse model (Lee et al., 2012b). Upregulation of GSH, GSH-Px, and SOD enhanced antioxidant capacity and reduced ROS generation and MDA content (Lee et al., 2012b; Zhang et al., 2021). Rg3 downregulates p53 and caspase-3 to inhibit apoptosis of kidney cells (Park et al., 2015; Zhang et al., 2021). For liver fibrosis, Rg3 decreased NF-κB to inhibit hepatic inflammation and fibrosis distribution in a nonalcoholic steatohepatitis mouse model (Lee et al., 2020b). In a thioacetamide (TAA)-induced hepatic fibrosis mouse model, Rg3 reduced inflammation-mediated autophagy and increased SOD, CAT, and GSH levels to improve liver function and attenuate fibrosis (Liu et al., 2020) (Table 2).

**TABLE 2:** Summary of Rg3 on liver and kidney injury.

Diseases	Model	Treatment	Outcome	Mechanism	Ref
**Liver injury**	Male ICR mice with injection of 250 mg/kg acetaminophen	10 and 20 mg/kg 20(R)-Rg3 for 7 days orally	ALT↓, AST↓, TNF-α↓, IL-1β↓, GSH↑, MDA↓, CYP2E1↓, 4-hydroxynonenal↓, Bax↓, Bcl-2↑, PI3K/Akt↑, IKKα↓, IKKβ↓, NF-κB↓	Inhibited the overexpression of CYP2E1 against oxidative stress, inhibited NF-κB to reduce inflammatory infiltration	Zhou et al. (2018)
**Liver injury**	C57BL/6J mice with intragastric 350 mg/kg acetaminophen	Oral 5, 10, and 20 mg/kg Rg3	ALT↓, AST↓, LDH↓, alkaline phosphatase↓, GSH↑, GSH-px↑, MDA↓, IL-1α↓, IL-1β↓, IL-5↓, IL-6↓, CCL3↓, CCL5↓, CCL11↓, TNF-α↓, caspase-1↓, NLRP3↓	Inhibited oxidative stress, inflammatory reaction and apoptosis through NLRP3	Gao et al. (2021)
**Liver and kidney injury**	Male Wistar rats with 5 mg/kg LPS	5 or 10 mg/kg 20(S)-Rg3 for 15 days orally	AST↓, ALT↓, CRE↓, NO_2_^−^/NO_3_^−^↓, TBA↓, NF-κB↓, COX-2↓, iNOS↓, HO-1↑	Inhibited NF-κB p65 to mitigate liver and kidney injury	Kang et al. (2007)
**Colon cancer and DDP-induced hepatotoxicity and nephrotoxicity**	LLC-RK1 cells, NCTC1469 cells and CT-26 cells	10–50 μM Rg3	BUN↓, CRE↓, ALT↓, AST↓, MDA↓, GSH↑, GSH-Px↑, SOD↑, ROS↓, Nrf2↓, HO-1↓	Enhanced the sensitivity of cisplatin, and ameliorate the kidney and liver damage against oxidative stress	Lee et al. (2012b)
	CT26 cells tumor-bearing nude mice	5 or 10 mg/kg Rg3			
**DDP-induced nephrotoxicity**	LLC-PK1 cells with 25 μM DDP	50, 100 and 250 μM Rg3	JNK↓, p53↓, caspase-3↓	Inhibited inflammation and apoptosis through downregulation of JNK and p53	Park et al. (2015)
**DDP-induced nephrotoxicity**	HK-2 and HepG2 cells with 4 μM DDP	1, 2 and 4 μM 20(R)-Rg3	BUN↓, CRE↓, MDA↓, CAT↑, SOD↑, GSH↑, ROS↓, iNOS↓, COX-2↓, p53↓, NF-κB↓	Downregulated NF-κB to improve oxidative stress against kidney damage	Zhang et al. (2021)
	ICR mice with 20 mg/kg DDP	10 and 20 mg/kg 20(R)-Rg3			
**Nonalcoholic steatohepatitis**	THP-1, HepG2, stellate cells, and hepatic LX2 cells with TGF-β, LPS or palmitate	0.01, 0.1, 1 and 10 μg/mL Rg3	α-SMA↓, collagen1↓, IL-1β↓, TNF-α↓, p-NF-κB↓	Decreased NF-κB for distribution of hepatic inflammation and fibrosis	Lee et al. (2020b)
	C57BL/6J mice with a methionine- and choline-deficient l-amino acid diet for 6 weeks	Oral 15 and 30 mg/kg Rg3			
**Hepatic fibrosis**	HSC-T6 and L02 cells with LPS	16 µM Rg3	ALT↓, AST↓, SOD↑, CAT↑, GSH↑, MDA↓, TGF-β1↓, α-SMA↓, p62↓, ATG5↓, ATG7↓, LC3b/LC3a↓, PI3K/Akt/mTOR↑	Reduced inflammation-mediated autophagy via upregulation of PI3K/Akt/mTOR	Liu et al. (2020)
	ICR mice with intraperitoneal injection TAA 150 mg/kg 4 weeks or 50 mg/kg TAA for 10 weeks	5 and 10 mg/kg Rg3 orally			

ICR, institute of cancer research; ALT, alanine aminotransferase; AST, aspartate aminotransferase; TNF-α, tumor necrosis factor-α; IL, interleukin; GSH, glutathione; MDA, malondialdehyde; CYP2E1, cytochrome P450 2E1; Bax, Bcl-2-associated X; Bcl-2, B-cell lymphoma-2; PI3K, phosphatidylinositol-3-kinase; IKK, inhibitory kappa kinase; NF-κB, nuclear factor-κB; LDH, lactate dehydrogenase; GSH-px, glutathione peroxidase; CCL, chemokine (C-C motif) ligand; LPS, lipopolysaccharide; NO, nitric oxide; CRE, creatinine; TBA, thiobarbituric acid; COX-2, cyclooxygenase-2; iNOS, inducible nitric oxide synthase; HO-1, heme oxygenase 1; DDP, cis-platinum; BUN, blood urea nitrogen; SOD, superoxide dismutase; ROS, reactive oxygen species; Nrf2, NF-E2-related factor 2; JNK, c-Jun N-terminal kinases; CAT, catalase; α-SMA, α-smooth muscle actin; TGF-β, transforming growth factor-β; TAA, thioacetamide; ATG, autophagy-related; LC3, light chain 3; mTOR, mammalian.

### 4.3 Neurological disorders

When neurons suffer oxidative stress, ROS cooperate with mitochondrial Ca^2+^ to increase the permeability of the mitochondrial transition pore (MPTP), resulting in mitochondrial swelling. This process leads to cellular inflammation, senescence, and apoptosis (Zhao et al., 2022). In an H_2_O_2_-induced mitochondrial suspension from the rat forebrain, 20(S)-Rg3 provided neuroprotection and inhibited mitochondrial swelling through suppression of ROS (Tian et al., 2009). Rg3 inhibited IL-6, IL-8, and monocyte chemotactic protein 1 (MCP-1) and ameliorated cellular senescence via inactivation of NF-κB and p38 in H_2_O_2_-treated human astrocyte CRT cells (Hou et al., 2017). The antioxidant effect of Rg3 mitigated neurotoxic effects associated with the prevention and treatment of neurodegeneration. Rg3 protected cortical cells from glutamate-induced nerve damage by enhancing SOD and GSH-px (Kim et al., 1998). Trimethyltin chloride-induced neurotoxicity of ICR mice promoted oxidative stress, inflammation, hippocampal neuron loss, and astrocytic activation in the mouse brain, which generates a clinical syndrome characterized by amnesia and complex seizures. Rg3 and Rh2 improved neurotoxicity via upregulation of PI3K/Akt and suppression of extracellular signal-regulated kinase (ERK) (Hou et al., 2018). In a mouse model of systemic inflammation mediated by LPS, 20(S)-Rg3 suppressed iNOS and COX-2 to attenuate inflammatory activation of the brain (Park et al., 2012).

Cognitive and memory impairments are closely associated with Alzheimer’s disease (AD). Chronic neuroinflammation of the entorhinal cortex and hippocampus leads to memory impairments (Lukiw and Bazan, 2000). The inhibition of COX-2 caused by Rg3 ameliorated LPS-induced learning and memory impairments in rats (Lee et al., 2013). Pathologically, the accumulation of abnormally folded amyloid-β (Aβ) and neuronal fiber aggregation lead to early cognitive impairment in AD. Rg3 reduced the neurotoxicity of Aβ42-treated BV-2 microglial cells. The mechanism suggested that Rg3 inhibited NF-κB p65 nuclear translocation to reduce the release of inflammatory factors and upregulated scavenger receptor type A (SRA) (Joo et al., 2008); this membrane protein recognizes pathological products and activates microglia to clear necrotic neurons (Bairamian et al., 2022). Bairamian *et al.* reported that Rg3 reduced ROS and Aβ deposition in a *Caenorhabditis elegans* model of AD (Tangrodchanapong et al., 2020). In addition, an *in vitro* study confirmed that regulation of inflammatory factors by Rg3 restored M2 activation and promoted Aβ uptake in microglia and neuronal cells (Ahn et al., 2021). In summary, Rg3 increases Aβ uptake to improve memory and cognitive function, which is associated with the inhibition of inflammatory factors through NF-κB and COX-2.

Parkinson’s disease (PD) is a neurodegenerative disorder with a decreased number of nigrostriatal neurons. Loss of nigrostriatal dopamine and α-synuclein aggregation correlates with the severity of PD. In a transgenic *Caenorhabditis elegans* model of PD, Rg3 suppressed apoptosis and enhanced antioxidant enzymes to prolong lifespan (Chalorak et al., 2021). Another study reported that Rg3 prolonged the latency of rotenone-induced PD mice through evaluation of pole, rotarod, and open field tests. The mice showed improved motor function and dopamine content in the striatum, suggesting that Rg3 enhances the expression of glutamate cysteine ligase (GCL) (Han et al., 2021), which catalyzes GSH synthesis to prevent oxidative damage and ROS generation (Forman et al., 2009). To sum up, Rg3 increases peroxidase activity to promote dopamine secretion and slow PD progression.

Cerebral ischemia is a common disease with severe consequences. The excessive accumulation of cytokines increases the adhesion of leukocytes to blood vessels, leading to endothelial damage and aggravating cerebral ischemia–reperfusion (I/R) injury (Candelario-Jalil et al., 2022). Rg3 attenuated IL-1β, IL-6, TNF-α, and ROS generation in a middle cerebral artery occlusion (MCAO) rat model. The mechanism suggests that Rg3 inhibits TLR4/MyD88 and SIRT1 to protect the brain from ischemic injury (Cheng et al., 2019).

Accelerated chronic inflammation and aging of nucleus pulposus cells (NPCs) lead to intervertebral disk degeneration (IDD) (Heathfield et al., 2008; Li et al., 2017). In TNF-α-treated NPCs, Rg3 improved oxidative stress status and extracellular matrix (ECM) metabolism by inhibiting NF-κB p65 activation in a dose-dependent manner (Chen et al., 2019). Spinal cord injury is a destructive spinal cord lesion that is more severe than IDD. 20(*S*)-Rg3 promoted the recovery of motor function and attenuated iNOS and COX-2 in a spinal cord injury rat model. (Kim et al., 2017).

In conclusion, Rg3 has a strong neuroprotective effect. When neurons are stimulated by peroxide, Rg3 scavenges free radicals, protects mitochondrial function, inhibits inflammatory factors, suppresses cell senescence. When neurons are exposed to toxic substances, Rg3 increases peroxidase activity to detoxify and activate PI3K/Akt to prevent apoptosis. The development of neurodegenerative and chronic diseases, including AD, PD, and stroke, is often accompanied by oxidative stress and inflammation (Zhao et al., 2022). These studies suggest that Rg3 inhibits cytokines, COX-2, ROS generation, and mitochondrial dysfunction and enhances peroxidase activity and microglial polarization to exert therapeutic effects on neurological disorders (Table 3).

**TABLE 3:** Summary of Rg3 in neurological disorders.

Diseases	Model	Treatment	Outcome	Mechanism	Ref
**Neurodegeneration**	Mixed cortical cells exposed to 50 μM glutamate for15 min, which were from old fetal SD rats	0.1 and 1 µM Rg3	LDH↓, SOD↑, H_2_O_2_↓, GSH-px↑, MDA↓, Nitrite↓, Ca^2+^ influx↓	Antioxidation on suppression of extensive neuronal death	Kim et al. (1998)
**Neuroprotection**	50 μM CaCl_2_ or 3 mM H_2_O_2_ induced mitochondrial suspension from rat forebrain	2–16 μM 20(S)-Rg3	Mitochondria swelling↓, ROS↓	Inhibited the opening of MPTP by free radical scavenging action	Tian et al. (2009)
**Brain aging**	35 μM H_2_O_2_ induced human astrocytic CRT cells; 150 μM H_2_O_2_ induced primary rat astrocytes	5 and 10 μg/mL Rg3	P53↓, P21↓, IL-6↓, IL-8↓, MCP-1↓, NF-κB↓, p38↓	Decreased senescence by suppressing NF-κB and p38 activation	Hou et al. (2017)
**Neuroinflammation**	C57BL/6 mice intraperitoneally injected with LPS (3 mg/kg)	20, and 30 mg/kg 20(S)-Rg3 orally	TNF-α↓, IL-1β↓, IL-6↓, iNOS↓, COX-2↓	Suppressed iNOS and COX-2 to attenuate microglia inflammation activation	Park et al. (2012)
**Neurotoxicity**	Primary rat astrocytes cultured with 1 μmol/L trimethyltin chloride	5 μg/mL Rg3	SOD↑, GSH-px↑, MDA↓, IL-1α/β↓, IL-6↓, TNF-α↓, Akt↑, ERK↓, cleaved caspase-3↓	Improved the oxidative stress and neuroinflammation through upregulation of PI3K/Akt and suppression of ERK	Hou et al. (2018)
	ICR mice intraperitoneal injection of 2 mg/kg trimethyltin chloride	20 mg/kg/day Rg3 injected intraperitoneally for 28 days			
**Learning and memory impairments**	Adult male SD rats with microinjection of 50 μg LPS into the lateral ventricles	10, 20 and 50 mg/kg Rg3 intraperitoneally for 21 days	Memory impairment↓	Decreased inflammatory mediators and COX-2 to repair the memory impairment	Lee et al. (2013)
			TNF-α↓, IL-1β↓, COX-2↓		
**Neurodegeneration**	5 μg/mL Aβ42 induced BV-2 microglial cells	10 μg/mL Rg3	IL-1β↓, IL-6↓, MCP-1↓, MIP-1↓, TNF-α↓, iNOS↓, NF-κB p65↓, SRA↑	Attenuated the inflammatory of microglia and neuroblastoma through suppression of NF-κB p65 nuclear translocation	Joo et al. (2008)
	50 ng/mL induced TNF-α Neuro-2a neuroblastoma cells	20 μg/mL Rg3			
**AD**	Transgenic *Caenditis elegans* model of AD	50 µM Rg3	Aβ deposit↓, ROS↓	-	Tangrodchanapong et al. (2020)
**AD**	20 ng/mL IFN-γ induced Neuro-2a murine neuroblastoma and HMO6 human microglial cells	5 μg/mL Rg3	IL-6↓, TNF-α↓, iNOS↓, Arg1↑, IL-10↑, SRA↑	Enhanced Aβ uptake through M2 microglial activation	Ahn et al. (2021)
**PD**	6-hydroxydopamine induced transgenic *Caenorhabditis elegans* model of PD	0.5, 1, 5, and 10 µM Rg3	Dopaminergic neurons↑, α-Synuclein↓, caspase-9↓, *Cat-2*↑, *Sod-3*↑	Suppressed apoptosis and enhanced antioxidant enzymes	Chalorak et al. (2021)
**PD**	C57BL/6J male mice intragastric administration with 30 mg/kg rotenone for 6 weeks	5, 10, or 20 mg/kg Rg3 intragastrically for 6 weeks	Dopamine↑, ROS↓, GCL↑	Improved motor function of PD mice through GCL	Han et al. (2021)
**Cerebral ischemia**	1 mmol/L CoCl_2_ induced PC12 cells	20 μg/mL Rg3	ROS↓, mitochondrial membrane↑, IL-1β↓, TNF-α↓, IL-6↓, TLR4/MyD88↓, NF-κB p65↓, SIRT1↑	Inhibited TLR4/MyD88 and activited SIRT1 to protected the brain from ischemic injury	Cheng et al. (2019)
	MCAO rat model	10 mg/kg Rg3 injected from the tail vein			
**IDD**	10 ng/mL TNF-α induced NPCs cells	25, 50 and 100 μg/mL Rg3	ROS↓, MDA↓, SOD↑, GSH↑, ECM metabolism↑ (MMP3↑, ADAMTS5↑, Aggrecan↓ and COL2A1↓), NF-κB p65↓	Improved oxidative stress, ECM metabolism and cell cycle through inhibiting NF-κB p65	Chen et al. (2019)
**Spinal cord injury**	Rats underwent laminectomy with spinal cord compression injury	Orally 10 or 30 mg/kg/day 20(*S*)-Rg3 for 14 days	Bax↓, Bcl-2↓, iNOS↓, COX-2↓, TNF-α, IL-1β, IL-6↓, Iba1↓	Promoted the recovery of motor function, reduced neuronal apoptosis and the activation of microglia via Iba1	Kim et al. (2017)

SD, rat, Sprague–Dawley rat; LDH, lactate dehydrogenase; SOD, superoxide dismutase; GSH-px, glutathione peroxidase; MDA, malondialdehyde; ROS, reactive oxygen species; MPTP, mitochondrial transition pore; IL, interleukin; MCP-1, monocyte chemotactic protein 1; NF-κB, nuclear factor-κB; LPS, lipopolysaccharide; TNF-α, tumor necrosis factor-α; iNOS, inducible nitric oxide synthase; COX-2, cyclooxygenase-2; ICR, institute of cancer research; ERK, extracellular signal-regulated kinase; MIP-1, macrophage inflammatory protein-1; SRA, scavenger receptor type A; AD, Alzheimer’s disease; IFN-γ, interferon-γ; Arg1, PD, Parkinson’s disease; GCL, glutamate cysteine ligase; MCAO, middle cerebral artery occlusion; TLR4/MyD88, Toll-like receptor 4/myeloid differentiation primary response 88; SIRT1, sirtuin 1; IDD, intervertebral disk degeneration; ECM, extracellular matrix; MMP, matrix metalloproteinases.

### 4.4 Cardiovascular diseases

In the early pathological processes of hypertension and atherosclerosis, chronic vascular inflammation and toxic metabolic compounds of lipid oxidation lead to vascular endothelial dysfunction and lipid deposition (Dasagrandhi et al., 2022). This process is accompanied by upregulation of vascular cell adhesion molecule-1 (VCAM-1) and intercellular adhesion molecule-1 (ICAM-1), and oxidized lipids and adhesion molecules mutually promote the plaque formation process of atherosclerosis (Blankenberg et al., 2003; Malekmohammad et al., 2019). In the presence of various inducers (e.g., LPS, NOD1 agonist, or ox-LDL), Rg3 downregulates ICAM-1 and VCAM-1 to inhibit endothelial dysfunction, which is related to anti-inflammatory action by the NF-κB pathway (Cho et al., 2014; Lee et al., 2020c; Geng et al., 2020). Rg3 inhibited THP-1-cell adhesion into human umbilical vein endothelial cells (HUVECs) by suppressing the NF-κB pathway *in vitro* (Cho et al., 2014). Oral Rg3 restrained the formation of atherosclerosis and inflammation in ApoE^−/−^ mice via the regulation of peroxisome proliferator-activated receptor γ (PPARγ)/focal adhesion kinase (FAK) (Geng et al., 2020).

NO is a significant factor in maintaining vascular homeostasis. Rg3 promoted NO release to increase thoracic aortic relaxation in rats, which had a therapeutic effect on hypertension (Kim et al., 1999; Nagar et al., 2016). The mechanism is related to the activation of eNOS or iNOS. Kim *et al.* suggested that iNOS was upregulated by NF-κB activation. Low-dose Rg3 promoted macrophage proinflammatory responses, including upregulation of IL-1 and TNF and NO release (Kim et al., 2003). Another study found that the activation and phosphorylation of eNOS mediated Rg3 treatment of hypertension. Rg3 activated estrogen receptor (ER)-dependent PI3K and AMP-activated protein kinase (AMPK) pathways to upregulate eNOS in endothelial cells (Hien et al., 2010). These studies suggest that Rg3 regulates vasodilatation by increasing the release of NO in hypertension.

Ginseng invigorates vitality in ethnic medicine, which is related to the regulation of heart function. Rg3 improved cardiac function and myocardial damage in rats with myocardial I/R surgery (Wang et al., 2015a; Zhang et al., 2016). In the treatment of myocardial injury, Rg3 reduced the secretion of IL-6, IL-1β, and TNF-α, inhibited lactic acid accumulation, improved the oxidative stress state, including upregulation of SOD content and downregulation of ROS accumulation, indicating that Rg3 is a SIRT1 agonist that downregulates the NF-κB pathway (Li et al., 2020a; Tu et al., 2020). Li *et al.* developed Rg3-loaded ROS-responsive polymeric nanoparticles, which have a lower therapeutic dose (Li et al., 2020a). In addition, Rg3 enhanced B-cell lymphoma-2 (Bcl-2) and inhibited p53, Bcl-2-associated X (Bax) and caspase-3 to resist apoptosis of cardiomyocytes *in vitro* and in SD rats with myocardial I/R surgery *in vivo* (Wang et al., 2015a; Zhang et al., 2016; Li et al., 2020a). Upregulation of Akt/eNOS for NO release by Rg3 protected against myocardial injury (Wang et al., 2015a). For hypertrophic cardiomyopathy, Rg3 alleviated oxidative stress, inflammation, and fibrosis via SIRT1/NF-κB in rats undergoing transverse aortic coarctation (TAC) surgery (Ren et al., 2021). Rg3 improved heart function and aortic ring endothelial function in adriamycin (ADM)-treated rats. The mechanism involved the activation of Nrf2/HO-1 to reduce ROS and increase eNOS levels (Wang et al., 2015b). The beneficial effects of Rg3 on cardiovascular disease are summarized in Table 4.

**TABLE 4:** Summary of Rg3 on cardiovascular diseases.

Diseases	Model	Treatment	Outcome	Mechanism	Ref
**Vascular inflammation**	HUVECs cells with the NOD1 agonist	10 μg/mL Rg3	NOD1↓, miR-139–5p↑, fibronectin↓, N-cadherin↓, VE-cadherin↓, smooth muscle-22α↓, NF-κB↓, IκBα↑, p65 (nucleus)↓	Inhibited inflammation and epithelial-mesenchymal transition through miR-139/NF-κB axis	Lee et al. (2020c)
**Vascular inflammation**	HUVECs with 1 μg/mL LPS stimulation and THP-1 macrophage cells	1, 10, 20 and 50 μM of Rg3	ICAM–1↓, VCAM–1↓, IκBα↑	Inhibited leukocyte adhesion into vascular wall and NF-κB pathway	Cho et al. (2014)
	Male C57BL/6 mice with 20 mg/kg LPS	20 mg/mL Rg3			
**Atherosclerosis**	200 μg/mL ox-LDL stimulated HUVECs, THP-1 cells	15 and 30 μM Rg3	VCAM-1↓, ICAM-1↓, MMP-2↓, MMP-9↓, NF-κB↓, MCP-1↓, IL-6↓, α-SMA↑, CD68↓, PPARγ↑, FAK↓	Upregulated PPARγ to repress FAK and inflammation	Geng et al. (2020)
	ApoE^−/−^ mice fed a high-fat diets	15 and 30 mg/kg Rg3 orally			
**Hypertension**	Thoracic aortas isolated from male SD rats	0.1 μg/mL Rg3	NO↑, cGMP↑, K^+^ channels↑	Increased endothelium derived NO production for relaxation of aortic rings	Kim et al. (1999)
**Hypertension**	Thoracic aortas of SD rats	100 mg/kg Rg3 orally for 5 days	iNOS↑, NO↑, NF-κB↑, IκBα↓, p-IκBα↑, p65 (nucleus)↑, IL-1↑, TNF-α↑	Activated NF-κB pathway to release iNOS for vasodilation	Kim et al. (2003)
	Raw264.7 cells	10 μg/mL Rg3			
**Hypertension**	ECV 304 cells	1, 3 and 10 μg/mL Rg3	NO↑, eNOS↑, p-eNOS↑, Akt↑, JNK↑, p38↑, AMPK↑, calmodulin-dependent protein kinase II↑	Activated eNOS through PI3K and AMPK pathway	Hien et al. (2010)
	Endothelium-intact aortic rings from thoracic aortas of SD rats	10 μg/mL Rg3			
**Myocardial I/R injury**	SD rats with myocardial I/R surgery	Intraperitoneal injection 60 mg/kg Rg3	LDH/CK↓, SOD↑, caspase-3↓, caspase-9↓, Bcl-2/Bax↑, p-eNOS↑, NO↑, p-Akt↑	Upregulated Akt/eNOS to reduced rat myocardial injury; Upregulated Bcl-2/Bax ratio to resist apoptosis	Wang et al. (2015a)
	1 mM CaCl_2_ induced primary neonatal rat cardiomyocytes	10 μM Rg3			
**Myocardial I/R injury**	Male SD rats with myocardial I/R surgery	5 or 20 mg/kg Rg3 intragastrically	Caspase-3↓, P53↓, Bax↓, Bcl-2↑, TNF-α↓, IL-1β↓	Ameliorated cardiac function through antioxidant and anti-apoptosis	Zhang et al. (2016)
**Myocardial I/R injury**	Male SD rats with myocardial I/R surgery	2.5 mg/kg Rg3-NPs injected into the heart	CK↓, CK-MB↓, LDH↓, FOXO3a↑, SIRT1↑, PPARγ; Antioxidant: ROS↓, SOD↑, MDA↓, Nrf1↑, Nrf2↑, HO-1↑, SOD1↑; Anti-inflammatory: C-reactive protein↓, IL-6↓, IL-1β↓, TNF-α1↑, p65 NFκ, p-IKBα↓	Increased FOXO3a/SIRT1 pathway to response to myocardial I/R injury	Li et al. (2020a)
	H9C2 cells	10 nM Rg3-NPs	Anti-fibrosis: TGF-β↓, p-Smad2/Smad2↓, MMP2↓, MMP9↓		
**Myocardial I/R injury**	Male SD rats with myocardial I/R surgery	30 mg/kg Rg3 intragastrically for 7 days	LDH↓, CK-MB↓, cardiac troponin I↓, TNF-α↓, IL-1β↓, IL-6↓, IL-10↑, SIRT1↑, p-p65/p65↓	Activated SIRT1 to inhibit the inflammatory response of myocardial infarction rats	Tu et al. (2020)
**Myocardial hypertrophy**	SD rats with transverse aortic coarctation surgery	30 mg/kg/day, intragastrically Rg3 for 14 days	Anti-fibrosis: myosin heavy chain↓, collagen I↓, TGF-β1↓	Alleviate oxidative stress, inflammation and fibrosis via SIRT1/NF-κB pathway	Ren et al. (2021)
			Inflammasome: NLRP3↓, ASC↓, caspase-1↓		
	Human cardiomyocytes AC16 and HCM cells cultured with 200 nM Angiotensin Ⅱ	10, 20 and 40 µM Rg3	Antioxidant: MDA↓, SOD↑, HO-1↑, Nrf2↑		
			SIRT1↑, NF-κB↓		
**ADM induced cardiotoxicity**	SD rats intraperitoneal injection of 15 mg/kg ADM	Rg3 intraperitoneal injection 10, 20, 40 mg/kg	ICAM-1↓, TGF-β↓, TIMP-1↓, VEGF↓, LDH↓, ROS↓, MDA↓, eNOS↑, endothelin-1↓, SOD↑, Nrf2↑, HO-1↑, Keap1↓	Activated Nrf2/HO-1 to improve cardiac function and aortic ring endothelial function	Wang et al. (2015b)
	CMEC cells isolated from neonatal rats with 1 μM ADM	Rg3 10 μM			

NOD1,nucleotide-binding oligomerization domain 1; NF-κB, nuclear factor-κB; IκB, inhibitor of NF-κB; LPS, lipopolysaccharide; ICAM–1, intercellular adhesion molecule-1; VCAM–1, vascular cell adhesion molecule-1; MMP, matrix metalloproteinases; MCP-1, monocyte chemotactic protein 1; IL, interleukin; α-SMA, α-smooth muscle actin; PPARγ, peroxisome proliferator-activated receptor-γ; FAK, focal adhesion kinase; SD, rat, Sprague–Dawley rat; NO, nitric oxide; cGMP′ 3′,5′-cyclic guanosine monophosphate; iNOS, inducible nitric oxide synthase; eNOS, endothelial nitric oxide synthase; TNF-α, tumor necrosis factor-α; JNK, c-Jun N-terminal kinases; AMPK, AMP-activated protein kinase; I/R, ischemia–reperfusion; LDH, lactate dehydrogenase; CK, creatine kinase; SOD, superoxide dismutase; Bcl-2, B-cell lymphoma-2; Bax, Bcl-2-associated X; NP, nucleus pulposus; ROS, reactive oxygen species; MDA, malondialdehyde; FOXO3a, Forkhead box O 3a; SIRT1, sirtuin 1; Nrf2, NF-E2-related factor 2; HO-1, heme oxygenase 1; TGF-β, transforming growth factor-β; ASC, apoptosis-associated speck-like protein; ADM, adriamycin; TIMP-1, tissue inhibitor of metalloproteinases 1; VEGF, vascular endothelial growth factor; Keap1, Kelch-like ECH-associated protein 1.

### 4.5 Diabetes and diabetic complications

Diabetes is a metabolic disease with increasing incidence worldwide. Oxidative stress promotes metabolic dysfunction and complications in diabetes (Bhatti et al., 2022). Rg3 exerts its therapeutic effect in the treatment of diabetes, diabetic nephropathy (DN), and dyslipidemia. In streptozotocin (STZ)-induced diabetes models, Rg3 reduced fasting blood glucose (FBG) and glycosylated protein (Kang et al., 2008; Li et al., 2021). iNOS, COX-2, and NF-кB p65 were decreased after Rg3 intervention (Kang et al., 2008; Kim et al., 2014). iNOS-derived overproduction of NO leads to the development of diabetes (Bahadoran et al., 2020). The NF-κB signaling cascade is the initial event in response to oxidative stress in diabetes (Archuleta et al., 2009). A study showed that Rg3 decreased urine protein associated with DN in a rat model via inhibition of NF-κB p65 (Zhou et al., 2020). Another study found that 20(S)-Rg3 ameliorated renal histopathological injury and dyslipidemia through the MAPK and NF-κB pathways in C57BL/6 mice with an intraperitoneal injection of STZ and a high-fat diet (HFD). At the same time, 20(S)-Rg3 improved oxidative stress status by diminishing the overproduction of MDA and enhancing serum SOD (Zhou et al., 2020). Guo *et al.* found that 20(S)-Rg3 skewed macrophages to the M2 phenotype and decreased the expression of TNF-α, IL-6, IL-10, and transforming growth factor-β (TGF-β) to mitigate atherosclerosis in diabetic apoE^−/−^ mice (Guo et al., 2018). For diabetic complications, inhibition of the apoptotic signaling pathway can protect renal tissue in DN. Rg3 protected renal tubular epithelial cells from apoptosis *in vitro* (Zhou et al., 2020). Li *et al.* suggested that Rg3 reduced Bax and cleaved caspase3 and enhanced the expression of Bcl-2 and Bcl-XL to inhibit apoptosis in the kidneys of DN mice (Li et al., 2021). In addition, Rg3 improved erectile function by upregulating SOD and protected nerve fibers from apoptosis in diabetic rats (Liu et al., 2015). These studies suggest that Rg3 is useful in treating diabetes and complications, attributable to its antioxidant, anti-inflammatory and anti-apoptosis effects (Table 5).

**TABLE 5:** Summary of Rg3 on diabetes and diabetic complications.

Diseases	Model	Treatment	Outcome	Mechanism	Ref
**Diabetes**	Islets isolated from BALB/c mice pancreas with high glucose concentration (16.7 mmol/L)	4 mmol/L Rg3 for 24, 48, and 72 h	NO↓, iNOS↓, PARP↓	Enhanced islet cell function and attenuated apoptosis through inhibiting iNOS and PARP	Kim et al. (2014)
**DN**	C57BL/6 mice with intraperitoneal injection of 100 mg/kg STZ and combined HFD	10 and 20 mg/kg 20(R)- Rg3 for 8 weeks orally	FBG↓, blood lipids↓, SOD↑, MDA↓, HO-1↑, TNF-α↓, IL-1β↑, p-JNK↓, p-ERK↓, p-p38↓, p-p65↓, cleaved caspase-3, 8↓, Bcl-2↑, Bcl-XL↑	Inhibited MAPK and NF-κB pathways to prevent DN	Li et al. (2021)
**Diabetes and DN**	Wistar rats with 50 mg/kg STZ injected intraperitoneally	Orally 5, 10, and 20 mg/kg/day 20(S)-Rg3	Serum glucose↓, TBA↓, glycosylated protein↓, NF-кB p65↓, COX-2↓, iNOS↓, 3-nitrotyrosine protein↓, N-methyl-d-aspartate↓	Improved the renal nitrosative stress of diabetes rats through NF-кB pathway	Kang et al. (2008)
**DN**	NRK-52E cells with palmitic acid	25 and 50 μM 20(S)-Rg3	Urine protein↓, TGF-β1↓, NF-κB p65↓, TNF-α↓	Inhibited NF-κB p65 to protect the renal tubular epithelial cell from apoptosis	Zhou et al. (2020)
	SD rats with intraperitoneal injection of 60 mg/kg STZ injected and combined HFD	10 mg/kg/day 20(S)- Rg3 for 12 weeks			
**Progress of atherosclerosis in diabetes**	THP-1 cells with 100 ng/mL phorbol-12-myristate-13 acetate and the bone marrow cells isolated from mice with 20 ng/mL macrophage colony-stimulating factor	25 μM 20(S)-Rg3	Blood glucose↓, blood lipids↓, TNF-α↓, IL-6↓, IL-10↑, TGF-β↑, PPARγ↓, iNOS↓, arginase-1↑, CD86↓, CD206↑	Skewed macrophages to the M2 phenotype to mitigate atherosclerosis in diabetic ApoE^−/−^ mice	Guo et al. (2018)
	ApoE^−/−^ mice 50 mg/kg STZ injected intraperitoneally	Intragastric 10 mg/kg 20(S)-Rg3			
**Diabetes and erectile function**	SD rats 60 mg/kg STZ injected intraperitoneally	Intragastric 10 and 100 mg/kg Rg3 for 3 months	Cleaved caspase‐3↓, Bcl‐2↑, Bcl‐xl↑, PECAM‐1↑, α-SMA↑, SOD↑, MDA↓	Improved erectile function through antioxidant effect	Liu et al. (2015)

NO, nitric oxide; iNOS, inducible nitric oxide synthase; PARP, poly (ADP-ribose) polymerase; DN, diabetic nephropathy; STZ, streptozotocin; HFD, high-fat diet; FBG, fasting blood glucose; SOD, superoxide dismutase; MDA, malondialdehyde; HO-1, heme oxygenase 1; TNF-α, tumor necrosis factor-α; IL, interleukin; JNK, c-Jun N-terminal kinases; ERK, extracellular signal-regulated kinase; Bcl-2, B-cell lymphoma-2; MAPK, mitogen-activated protein kinase; NF-κB, nuclear factor-κB; TBA, thiobarbituric acid; COX-2, cyclooxygenase-2; SD, rat, Sprague–Dawley rat; TGF-β, transforming growth factor-β; PPARγ, peroxisome proliferator-activated receptor-γ; α-SMA, α-smooth muscle actin.

### 4.6 Cancer

Rg3-rich extracts of ginseng upregulated NO production to activate an immune response in LPS-treated Raw 264.7 cells and inhibited the proliferation of HUVECs (Lee et al., 2009). Interestingly, Kim *et al.* observed that Rg3 inhibited the differentiation and migration of endothelial progenitor cells through suppression of vascular endothelial growth factor (VEGF)/Akt/eNOS signaling, suggesting an antiangiogenic effect (Kim et al., 2012). However, the study did not detect the release of NO. In addition, a red ginseng preparation with Rg3-fortified facilitated NO release for the proliferation of splenocytes and inhibition of tumor growth in lung cancer tumor-bearing mice (Park et al., 2011). These findings suggest that the promotion of NO release by Rg3 inhibits angiogenesis and tumor proliferation; however, the synthesis pathway of NO production requires further study.

Oxidative stress status and tumor therapy need to be analysed from two aspects. ROS accumulation in tumor tissue induces tumor cell apoptosis (e.g., ferroptosis) (Liang et al., 2019). On the other hand, free radicals derived from tumor cells promote DNA damage and mutation to cause tumorigenesis and tumor metastasis (Prasad et al., 2017). Graphene oxide nanoparticle-loaded Rg3 promoted apoptosis and enhanced ROS generation to enhance photodynamic therapy of osteosarcoma (Lu et al., 2021). Rg3 nanoparticles (Rg3-NPs) promoted apoptosis and improved oxidative stress states against Ehrlich solid tumor growth in mice (El-Banna et al., 2022). Regarding tumorigenesis, abnormal expression of COX-2 and proinflammatory cytokines is associated with skin carcinogenesis (Moon et al., 2020). The topical application of Rg3 inhibited COX-2 and decreased skin carcinogenesis through the downregulation of the transcription factors NF-κB and activator protein-1 (AP-1) (Keum et al., 2003).

### 4.7 Other oxidative stress-related diseases

The aging process is accompanied by impairment of free radical scavenging and oxidative stress damage (Papaconstantinou, 2019). Rg3 increases mitochondrial biogenesis and enhances antioxidation to reduce ROS against cell senescence (Hong et al., 2020). Reduction of ROS by Rg3 protected keratinocytes from ultraviolet irradiation (Lim et al., 2014). In a d-galactose-induced aging mouse model, Rg3 inhibited CYP2E1 and MDA to protect against aging-related hepatic and renal dysfunction (Li et al., 2020b). Rg3 protected the gastric mucosa and inhibited gastric ulcer formation. Rg3 reduced iNOS-reducing NO release and the accumulation of free radicals in gastric mucosa mediated by alcohol; Rg3 also promoted the expression of epidermal growth factor (EGF) for epithelial proliferation and tissue repair (Zhang et al., 2019). Nanoparticles loaded with Rg3 could increase gastric ulcer treatment efficacy (Cao et al., 2022). Rg3 improved mitochondrial biogenesis and downregulated ROS by activating SIRT1 in TNF-α-treated chondrocytes, which could serve as an adjunct therapy for arthritis (Ma et al., 2021). Rg3 attenuated aluminum-induced osteoporosis by enhancing GSH and SOD activity (Song et al., 2020). Cyclophosphamide is an alkylating agent used to treat hematologic malignancies; however, it is associated with bone marrow toxicity. Rg3 inhibited CP-induced DNA damage by improving oxidative stress states *in vivo* (Zhang et al., 2008). In TNF-α-treated human chondrocytes, Rg3 inhibited mitochondrial ROS production and enhanced ATP content, which has potential as a therapeutic remedy for muscle weakness and atrophy (Lee et al., 2019). Several studies found that Rg3 inhibited the release of inflammatory factors and downregulated VEGF expression during wound healing, which is a potential treatment for hypertrophic scars (Cheng et al., 2013; Cheng et al., 2014; Ma et al., 2020).

## 5 Overview of the therapeutic potential of ginsenoside Rg3

Rg3 has potent antioxidant and anti-inflammatory effects, indicating that it might have a potential therapeutic role in acute organ injuries such as acute lung injury, acute liver and kidney injury, etc. Rg3 alleviates inflammatory infiltration of lung tissue and inhibits IL release in lung tissue in mouse and rat models of ALI (Yang et al., 2018). In an OVA-induced asthma mouse model, Rg3 ameliorates goblet cell hyperplasia and eosinophil infiltration (Huang et al., 2021). For liver and kidney injury caused by external factors, Rg3 significantly improves liver function, mainly by inhibiting the abnormal elevation of aminotransferases and overexpression of CYP2E1; meanwhile, Rg3 improves acetaminophen-induced focal centrilobular necrosis and pericentral venous degeneration of liver tissue (Zhou et al., 2018; Gao et al., 2021). In addition, Rg3 inhibits the expression of α-SMA and collagen I during the fibrosis process of nonalcoholic steatohepatitis and hepatic fibrosis (Lee et al., 2020b; Liu et al., 2020). In DDP-mediated nephrotoxicity, Rg3 increases kidney tissue GSH expression, inhibits MDA levels, and downregulates serum BUN and CRE to protect renal function (Lee et al., 2012b; Zhang et al., 2021).

Rg3 can improve cardiac function, reduce markers of cardiac damage, suppress neurocyte aging, and reduce memory impairment in rats (Lee et al., 2013; Hou et al., 2017), indicating that Rg3 might also have the potential to become a promising therapeutic agent for chronic diseases, including cardiovascular diseases, cerebrovascular diseases, diabetes and cancers, etc. Rg3 enhanced Aβ uptake through M2 microglial activation in AD (Ahn et al., 2021). Rg3 prolonged the latency of rotenone-induced PD mice through evaluation of pole, rotarod, and open field tests (Han et al., 2021). The above effects can improve neurological damage, memory deterioration and cognitive impairment, which alleviates the progression of neurodegenerative diseases. In cardiovascular diseases, vascular inflammation and lipid accumulation are reduced after Rg3 intervention (Lee et al., 2020c; Geng et al., 2020). Several studies concluded that Rg3 activated eNOS for vasodilation in the treatment of hypertension (Kim et al., 2003; Hien et al., 2010). Rg3 enhanced left ventricular ejection fraction (EF%) to improve cardiac function in rats with myocardial I/R surgery. Rg3 ameliorated myocardial infarction-related serum markers, including creatine kinase (CK), CK-MB, and lactate dehydrogenase (LDH) (Wang et al., 2015a; Zhang et al., 2016). In metabolic diseases, Rg3 reduces FBG, glycosylated protein and blood lipids in diabetic rats (Kang et al., 2008; Li et al., 2021). Rg3 improves CRE and urine protein elevation in DN mice by increasing antioxidant activity and reducing renal inflammation (Zhou et al., 2020; Li et al., 2021). Rg3 inhibited the proliferation and metastasis of various cancer cells, such as liver cancer cells, intestinal cancer cells, osteosarcoma cells, etc. (Sun et al., 2017). Either Rg3 alone or Rg3 in combination with DDP suppressed the growth of colon tumor in nude mice (Lee et al., 2012b). Treatment with Rg3 decreased the tumor size of Ehrlich solid tumor in mice (El-Banna et al., 2022).

In summary, increasing evidence has shown that Rg3 has therapeutic potential for the treatment of acute organ injuries and chronic diseases, which is worthy of further research.

## 6 Discussion

**FIGURE 2:**
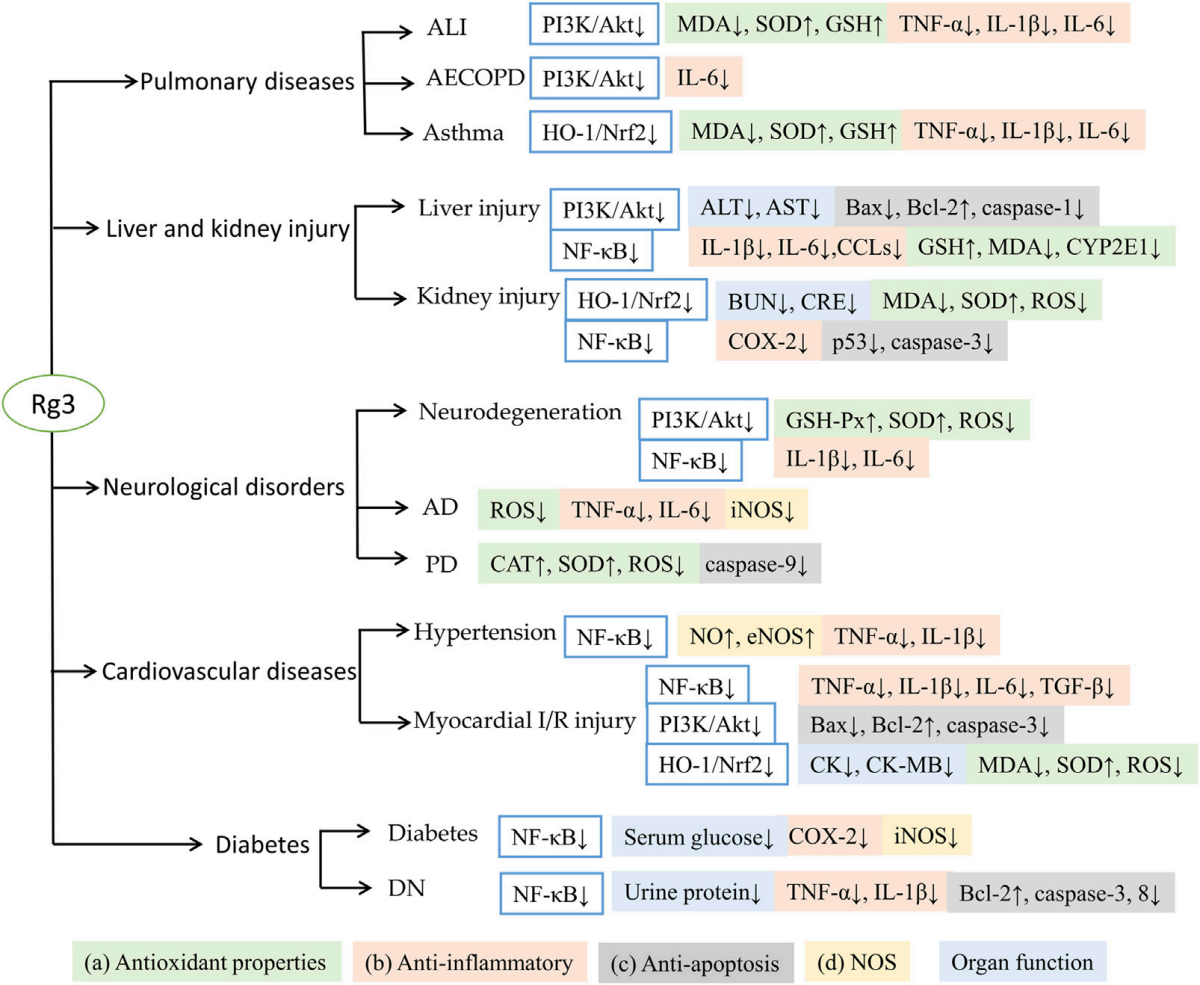
Summary of Rg3 against different diseases with antioxidant and anti-inflammatory effects.

The antioxidant and anti-inflammatory effects of Rg3 have been attributed to 1) antioxidant properties, including inhibition of ROS accumulation, promotion of peroxidase activity, and reduction of MDA content; 2) anti-inflammatory properties, inhibition of TNF-α, interleukins, chemokine ligands, suppression of NLRP3 formation and COX-2 expression; 3) regulation of apoptosis: downregulating the expression of p53, caspases and Bax/Bcl-2 ratio to protect tissue injury and apoptosis; and 4) regulation of NOS, inhibition of iNOS to reduce oxidative stress and upregulation of eNOS to protect vascular endothelial function. These antioxidant and anti-inflammatory activities of Rg3 help to maintain or improve organ functions, such as reducing neuroinflammation, attenuating neurotoxicity, protecting myocardial, hepatic and renal functions (Figure 2).

**FIGURE 3:**
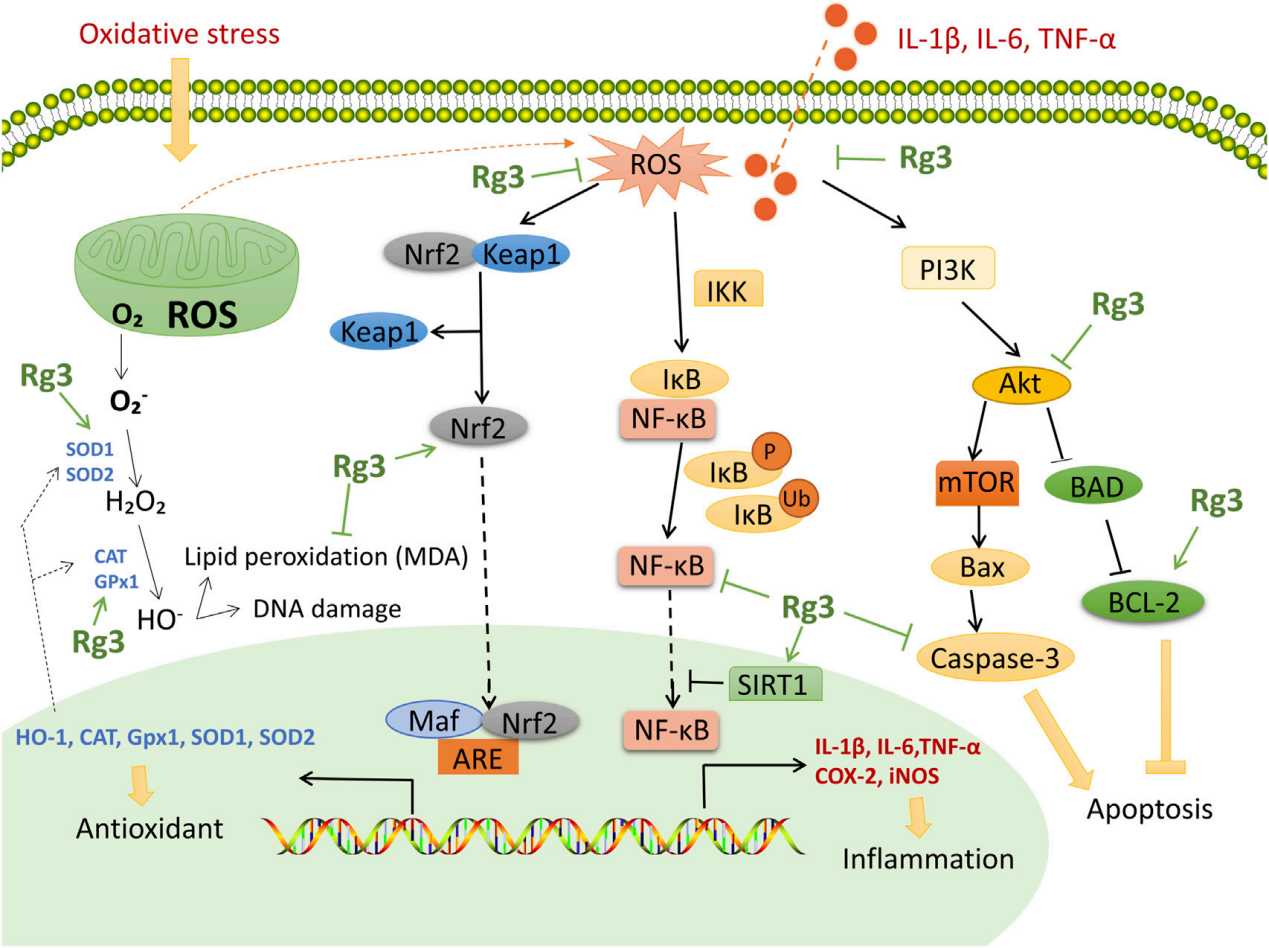
Functional mechanisms and targets of Rg3 on antioxidant and anti-inflammatory effects. Rg3 upregulates Nrf2 to enhance antioxidase activity. Rg3 inhibits the NF-κB pathway and downstream COX-2 to ameliorate inflammation and oxidative stress. Rg3 activates SIRT1 to inhibit the NF-κB pathway. Rg3 protects tissue from apoptosis through the PI3K/Akt pathway.

These effects are related to several potential mechanisms of action: (Lee et al., 2020a): inhibition of the NF-κB pathway decreases downstream COX-2 to ameliorate inflammation and oxidative stress; (Hyun et al., 2022); the PI3K/Akt pathway regulates the process of inflammation and apoptosis; and (Lee et al., 2017a) upregulation of the Nrf2/HO-1 pathway is an important pathway to reduce oxidative stress. Therefore, the key mechanism of action of Rg3 is shown in Figure 3. However, the content of these studies tends to be homogeneous, and there is a lack of Rg3 target screening based on high-throughput methods. In the future, we look forward to clinical research to explore the antioxidant activity of Rg3 and in-depth research into the critical targets of Rg3 antioxidant and anti-inflammatory activity.

## 7 Conclusion

Increasing evidence supports that ginsenoside Rg3 has remarkable anti-inflammatory and antioxidant effects on animal and cell models with various diseases, such as pulmonary, neurological, cardiovascular, metabolic diseases, cancer, and liver and kidney injury, and the multitarget and multipathway molecular mechanisms of action of Rg3 have been gradually deciphered. Rg3 may be a promising candidate drug for the treatment of diseases with inflammatory and oxidative stress conditions and is worthy of further research and development.
